# Tobacco smoking, body mass index, hypertension, and kidney cancer risk in central and eastern Europe

**DOI:** 10.1038/sj.bjc.6604761

**Published:** 2008-10-28

**Authors:** P Brennan, O van der Hel, L E Moore, D Zaridze, V Matveev, I Holcatova, V Janout, H Kollarova, L Foretova, N Szeszenia-Dabrowska, D Mates, N Rothman, P Boffetta, W-H Chow

**Affiliations:** 1 International Agency for Research on Cancer (IARC), Lyon, France; 2 Division of Cancer Epidemiology and Genetics, National Cancer Institute, NIH, DHHS, MD, USA; 3 Institute of Carcinogenesis, Cancer Research Center, Moscow, Russia; 4 Department of Urology, NN Blokhin Cancer Research Centre, Moscow, Russia; 5 Charles University in Prague, First Faculty of Medicine, Prague, Czech Republic; 6 Department of Preventive Medicine, Faculty of Medicine, Palacky University, Olomouc, Czech Republic; 7 Department of Cancer Epidemiology & Genetics, Masaryk Memorial Cancer Institute, Brno, Czech Republic; 8 Department of Epidemiology, Institute of Occupational Medicine, Lodz, Poland; 9 Institute of Public Health, Bucharest, Romania

**Keywords:** BMI, eastern Europe, hypertension, kidney cancer, smoking

## Abstract

In a case–control study of kidney cancer in four central European countries, with 1097 incident cases and 1476 controls, we found an increased risk for self-reported hypertension and for obesity. Additional unknown risk factors are likely to be responsible for the high rates of kidney cancer in this region.

Kidney cancer accounts for 1.9% of all malignancies, with approximately 189 000 new cases diagnosed globally each year and the incidence varying more than 10-fold around the world, with the highest rates generally observed in central European countries and among blacks in the United States (Murai and Oya, 2004). Smoking is an established risk factor, although the increase in risk is moderate (IARC, 2004). Other possible risk factors are hypertension and obesity, with potentially differing risks for men and women (Shapiro *et al*, 1999; Bergstrom *et al*, 2001). To examine the risk factors for kidney cancer in central Europe, we conducted a large multicenter case–control study in Czech Republic, Poland, Russia, and Romania. We present here data on the role of smoking, hypertension, and body mass index (BMI) in this high-risk population.

## Materials and methods

This hospital-based case–control study was conducted in seven centres (Moscow (Russia), Bucharest (Romania), Lodz (Poland), and Prague, Olomouc, Ceske Budejovice, and Brno (Czech Republic)). A total of 1097 newly diagnosed, histologically confirmed, renal parenchymal cancers (ICD-O-2 code C64) between 20 and 79 years of age were recruited during August 1999 and January 2003. Trained medical staff reviewed medical records to extract relevant diagnostic information, including date and method of diagnosis, histologic type, tumour location, stage, and grade. Eligible controls (*n*=1476) were admitted to the same hospitals as the cases for conditions unrelated to smoking or genitourinary disorders (except for benign prostatic hyperplasia) between August 1998 and March 2003. No single disease made up more than 20% of the control group. Both cases and controls had to be residents of the study areas for at least 1 year. The response rate for cases ranged from 90 to 98.6% and for the controls, 90.3–96.1%.

Trained interviewers used standardised questionnaires at all centres to elicit information on demographic background, smoking, alcohol drinking, dietary practices, height, weight, medical history, family history of cancer, residential history, and occupational history.

Smoking status (never smoker, former smoker, current smoker) was defined as status 2 years before interview. Packyears were used as a measure of cumulative tobacco smoking and were calculated by the number of cigarettes smoked per day multiplied by years of smoking and divided by 20. History of hypertension was self-reported and a positive history was restricted to patients who reported being treated for hypertension. The weight in kilograms was ascertained for 2 years before the interview. Body mass index was calculated by dividing the weight by the square of the height in metres.

Kidney cancer risks were estimated by odds ratios (ORs) and 95% confidence intervals (CIs) using logistic regression analysis, with adjustment for age (5-years interval), smoking (current, former, and never smokers), BMI in five categories (<25, 25–27.4, 27.5–29.9, 30–34.9, ⩾35), history of hypertension treatment (no *vs* yes), and country, where appropriate. Heterogeneity tests were used to evaluate differences among countries by including country–exposure interaction terms in the logistic models, and likelihood ratio tests to evaluate the statistical significance of the interaction terms.

## Results

The study population consisted of 1097 kidney cancer cases (648 men and 449 women) and 1476 controls (952 men and 524 women) (Tables 1 and 2). Among cases, tumour stage at diagnosis was similar for both sexes, with 9.2% of men and 6.9% of women having M1 stage and 10.3% of men and 8.8% of women having ⩾N1 stage, 83% of cases were clear cell carcinoma and 6% papillary carcinoma.

Smoking was not associated with an increased risk of kidney cancer, and no dose–response was seen with increasing levels of cigarette consumption. The findings were similar for all patients combined or in men and women separately. In addition, no significant heterogeneity between the four countries was observed.

Increasing BMI was positively associated with kidney cancer overall (test for linear trend, *P*=0.011; Table 3). Compared to those with BMI <25, the OR among those with BMI 30–35 was 1.38 (95% CIs: 1.09, 1.75), and among those with BMI ⩾35 was 1.29 (95% CIs: 0.88, 1.89). The excess risks, however, were restricted to men, risk nearly doubling among those in the highest category of BMI (OR=1.72; (95% CIs: 1.01, 2.94)). For women, no increase in risk was observed either overall or in any of the four countries separately. Approximately 14% of cases were estimated to be attributable to a BMI of greater than 25 (95% CI 3–23%).

An increased risk was observed for patients who reported having been treated for hypertension more than 2 years before interview compared to those who did not (OR=1.25 (95% CIs: 1.06, 1.49)) (Table 4). The effect was more prominent among women (OR=1.47 (95% CIs: 1.11, 1.95)) than men. The results were largely unaltered when hypertension was defined as ever having been diagnosed with hypertension regardless of treatment status. Approximately 9% of cases were estimated to be attributable to hypertension (95% CIs 2–16%).

Our analysis included all histological types of carcinoma of the renal parenchyma, including 17% of cases who had an histology other than clear cell type, excluding these cases had little influence on the results.

## Discussion

This study provides evidence that increased BMI and a history of hypertension are risk factors for kidney cancer in high-risk areas of central and eastern Europe, countries in which data on kidney cancer have been limited. The increased risk associated with BMI was more pronounced among men, while the increased risk for history of hypertension was more pronounced among women.

Our findings of increasing risks for kidney cancer with increasing BMI agree with most other studies. A recent evaluation by an IARC working group on the effects of body weight and adiposity showed that all but one of the 19 reviewed studies found a more than two-fold increase in kidney cancer risk among obese men and women compared with those of normal weight (IARC, 2002). In addition, two recent cohort studies confirmed a role for BMI (Bjorge *et al*, 2004; van Dijk *et al*, 2004). A potential mechanism by which obesity may increase kidney cancer risk involves increased levels of insulin-like growth factor (IGF) or lipid peroxidation. Increasing BMI is associated with elevated levels of fasting serum and free IGF-I among both men and women (Frystyk *et al*, 1995). Insulin-like growth factor-I stimulates cell proliferation and inhibits apoptosis, which could have a profound impact on tumour growth (Yu and Rohan, 2000).

An association between hypertension and kidney cancer has also been previously reported (McLaughlin *et al*, 2006). Hypertension is hypothesised to cause renal damage directly or cause metabolic or functional changes within the renal tubules, thus increasing the kidney's susceptibility to carcinogens or promoting agents (Cowley and Roman, 1996). The relationship between kidney cancer and hypertension is complex. McCredie and Stewart (1992) showed that kidney cancer in hypertensive patients was related to the duration of hypertension, suggesting that hypertension contributes is aetiologically relevant rather than being a consequence of the tumour (McCredie and Stewart, 1992). Also, in this connection, cohort studies may stratify by follow-up time, several finding that have found that the effect of hypertension is the same in the first years of follow-up as later (Fraser *et al*, 1990; Coughlin *et al*, 1997; Chow *et al*, 2000). These findings support the hypothesis that hypertension is a risk factor for kidney cancer. Further, an increased risk has been reported with mild hypertension that would usually go untreated, and a decreasing risk of among those who have experienced lowered blood pressure levels over time (Chow *et al*, 2000).

Smoking is an established risk factor for kidney cancer, although the increase in risk is weak (IARC, 2004). In a comprehensive meta-analysis, we have recently estimated that the increased risk for ever smokers compared to never smokers was 38% (RR=1.38 (95% CI: 1.28, 1.49) (Hunt *et al*, 2005)). There was also a strong dose-dependent increase with increasing tobacco consumption. However, the OR for smoking obtained from the meta-analysis in hospital-based studies was 1.17 (95% CIs: 1.03, 1.34), whereas in population-based studies, it was 1.49 (95% CIs: 1.34, 1.66). Our current study had a 97% power of detecting an increased risk of 40% associated with smoking, although only about a 50% power of detecting a 20% increase in risk. One possible conclusion is that smokers were over-represented in hospital controls resulting in a reduced power to detect a real effect of smoking on kidney cancer. We did compare the smoking prevalence between the major control groups and did not detect any heterogeneity, although a small amount of bias could not be excluded.

Our study confirms that BMI and a history of hypertension are risk factors for kidney cancer in central Europe, a region with the highest incidence currently reported, but these are unlikely to explain fully the high incidence, indicating that important causes remain to be discovered.

## Figures and Tables

**Table 1:** Descriptive characteristics of cases and controls

	**Men**				**Women**			
	**Cases**		**Controls**		**Cases**		**Controls**	
**Variables**	***N***	**%**	***N***	**%**	***N***	**%**	***N***	**%**
*Country*								
Romania	63	9.7	109	11.4	32	7.1	51	9.7
Poland	56	8.6	112	11.8	43	6.6	86	16.4
Russia	163	25.4	305	32.0	154	34.3	158	30.2
Czech Republic	366	56.4	426	44.8	220	49.0	229	43.7
Total	648	100	952	100	449	100	524	100
								
*Age (years)*								
<40	25	4.1	25	2.6	14	3.1	18	3.4
40–49	91	14.0	152	16.0	53	11.8	60	11.5
50–59	227	35.0	320	33.6	122	27.2	153	29.2
60–69	187	28.9	289	30.4	150	33.5	170	32.4
>70	118	18.0	166	17.4	110	24.4	123	23.5
								
*Tumour stage*								
*T-stage*								
T1	117	18.8			90	20.5		
T2	275	44.1			209	47.5		
T3	210	33.6			126	28.6		
T4	22	3.5			15	3.4		
								
*M-stage*								
M0	486	90.8			363	93.1		
M1	49	9.2			27	6.9		
								
*N-stage*								
N0	505	89.7			371	91.2		
N1	31	5.5			24	5.9		
N2	24	4.3			10	2.4		
N3	3	0.5			2	0.5		
								
*Histology*								
(Clear cell) Renal cell carcinoma	538	83.0			370	82.4		
Papillary renal cell carcinoma	51	7.9			20	4.5		
Other	59	9.1			59	13.1		

**Table 2:** Odds ratio of kidney cancer for tobacco smoking

	**Men**				**Women**				**Total**			
	**Cases**	**Controls**	**OR^a^**	**95% CI**	**Cases**	**Controls**	**OR^a^**	**95% CI**	**Cases**	**Controls**	**OR^a^**	**95% CI**
**Smoking**	***N***	***N***			***N***	***N***			***N***	***N***		
Never smokers	170	232	1.00	Ref^b^	340	368	1.00	Ref	510	600	1.00	Ref
Former smokers	204	302	0.89	0.68, 1.17	47	51	1.07	0.69, 1.67	251	353	0.88	0.71, 1.11
Current smokers	273	416	0.99	0.76, 1.29	60	105	0.70	0.48, 1.02	333	521	0.87	0.71, 1.07
												
Never smokers	170	232	1.00	Ref	340	368	1.00	Ref	510	600	1.00	Ref
<17 packyears	150	221	0.94	0.70, 1.26	62	97	0.76	0.52, 1.11	212	318	0.85	0.67, 1.06
17–31 packyears	161	234	1.00	0.75, 1.34	32	39	0.99	0.59, 1.65	193	273	0.95	0.74, 1.21
>31 packyears	165	261	0.89	0.67, 1.19	13	19	0.85	0.40, 1.78	178	280	0.85	0.66, 1.09
			*P*-value for trend 0.49				*P*-value for trend 0.34				*P*-value for trend 0.19	

a Adjusted for age, body mass index, history of hypertension and country.

b Ref=reference category.

**Table 3:** Odds ratio of kidney cancer for BMI

	**Men**				**Women**				**Total**			
	**Cases**	**Controls**	**OR^a^**	**95% CI**	**Cases**	**Controls**	**OR^a^**	**95% CI**	**Cases**	**Controls**	**OR^a^**	**95% CI**
**BMI**	**N**	***N***			***N***	***N***			***N***	***N***		
<25	191	363	1.00	Ref^b^	136	169	1.00	Ref	327	532	1.00	Ref
25–27.5	166	248	1.19	0.91, 1.56	87	115	0.86	0.60, 1.25	253	363	1.09	0.88, 1.35
27.5–29.99	125	167	1.32	0.98, 1.79	98	90	1.16	0.80, 1.70	223	257	1.31	1.04, 1.65
30–35	133	139	1.70	1.25, 2.31	98	111	0.95	0.66, 1.38	231	250	1.38	1.09, 1.75
35+	32	32	1.72	1.01, 2.94	30	37	0.85	0.49, 1.48	62	69	1.29	0.88, 1.89
			*P*-value for trend 0.001				*P*-value for trend 0.68				*P*-value for trend 0.011	

a Adjusted for age, smoking, history of hypertension and country.

b Ref=reference category.

**Table 4:** Odds ratio of kidney cancer for history of hypertension

	**Men**				**Women**				**Total**			
	**Cases**	**Controls**	**OR^a^**	**95% CI**	**Cases**	**Controls**	**OR^a^**	**95% CI**	**Cases**	**Controls**	**OR^a^**	**95% CI**
**History of treated hypertension**	***N***	***N***			***N***	***N***			***N***	***N***		
No	385	607	1.00	Ref^b^	215	299	1.00	Ref	600	906	1.00	Ref
Yes	262	344	1.12	0.90, 1.40	234	225	1.47	1.11, 1.95	496	569	1.25	1.06, 1.49

a Adjusted for age, smoking, BMI and country.

b Ref=reference category.

## References

[bib1] Bergstrom A, Hsieh CC, Lindblad P, Lu CM, Cook NR, Wolk A (2001) Obesity and renal cell cancer – a quantitative review. Br J Cancer 85: 984–990 11592770 10.1054/bjoc.2001.2040 PMC2375100

[bib2] Bjorge T, Tretli S, Engeland A (2004) Relation of height and body mass index to renal cell carcinoma in two million Norwegian men and women. Am J Epidemiol 160: 1168–1176 15583369 10.1093/aje/

[bib3] Chow WH, Gridley G, Fraumeni Jr JF, Järvholm B (2000) Obesity, hypertension, and the risk of kidney cancer in men. N Engl J Med 343: 1305–1311 11058675 10.1056/NEJM200011023431804

[bib4] Coughlin SS, Neaton JD, Randall B, Sengupta A (1997) Predictors of mortality from kidney cancer in 332 547 men screened for the multiple risk factor intervention trial. Cancer 79: 2171–2177 9179064

[bib5] Cowley Jr AW, Roman RJ (1996) The role of the kidney in hypertension. JAMA 275: 1581–1589 8622250

[bib6] Fraser GE, Phillips RL, Beeson WL (1990) Hypertension, antihypertensive medication and risk of renal carcinoma in California Seventh-Day Adventists. Int J Epidemiol 19: 832–838 2084009 10.1093/ije/19.4.832

[bib7] Frystyk J, Vestbo E, Skjaerbaek C, Mogensen CE, Orskov H (1995) Free insulin-like growth factors in human obesity. Metabolism 44: 37–44 7476310 10.1016/0026-0495(95)90219-8

[bib8] Hunt JD, van der Hel OL, McMillan GP, Boffetta P, Brennan P (2005) Renal cell carcinoma in relation to cigarette smoking: meta-analysis of 24 studies. Int J Cancer 114: 101–108 15523697 10.1002/ijc.20618

[bib9] IARC (2002) Handbooks of Cancer Prevention Vol. 6 Weight Control and Physical Activity. IARC Press: Lyon, France

[bib10] IARC (2004) IARC Monographs on the Evaluation for the Carcinogenic Risk of Chemicals to Humans: Tobacco Smoke and Involuntary Smoking. IARC press: Lyon PMC4781536 15285078

[bib11] McCredie M, Stewart JH (1992) Risk factors for kidney cancer in New South Wales, Australia. II. Urologic disease, hypertension, obesity, and hormonal factors. Cancer Causes Control 3: 323–331 1617119 10.1007/BF00146885

[bib12] McLaughlin JK, Lipworth L, Tarone RE, Blot WJ (2006) Renal cancer. In: ‘Cancer Epidemiology and Prevention’, Section: ‘Cancer by Tissue of Origin’ Schottenfeld D, Fraumeni Jr JF (eds) pp 1087–1100. Oxford University Press: Oxford, UK

[bib13] Murai M, Oya M (2004) Renal cell carcinoma: etiology, incidence and epidemiology. Curr Opin Urol 14: 229–233 15205579 10.1097/01.mou.0000135078.04721.f5

[bib14] Shapiro JA, Williams MA, Weiss NS, Stergachis A, LaCroix AZ, Barlow WE (1999) Hypertension, antihypertensive medication use, and risk of renal cell carcinoma. Am J Epidemiol 149: 521–530 10084241 10.1093/oxfordjournals.aje.a009848

[bib15] van Dijk BA, Schouten LJ, Kiemeney LA, Goldbohm RA, van den Brandt PA (2004) Relation of height, body mass, energy intake, and physical activity to risk of renal cell carcinoma: results from the Netherlands Cohort Study. Am J Epidemiol 160: 1159–1167 15583368 10.1093/aje/kwh344

[bib16] Yu H, Rohan T (2000) Role of the insulin-like growth factor family in cancer development and progression. J Natl Cancer Inst 92: 1472–1489 10995803 10.1093/jnci/92.18.1472

